# Supplementary material for: All-at-once RNA folding with 3D motif prediction framed by evolutionary information
Source: Nat Methods. 2025 Oct 3;22(10):2094–106. doi: 10.1038/s41592-025-02833-w (PMC12510878; doi:10.1038/s41592-025-02833-w)
Supplement: Supplementary file 3 — Source code to run CaCoFold-R3D. Data and results. [file 41592_2025_2833_MOESM3_ESM.gz › supplemental_material/results/CaCoFold-R3D/CaCoFold-R3D_Rfam/Rfam.rscape.times.pdf]

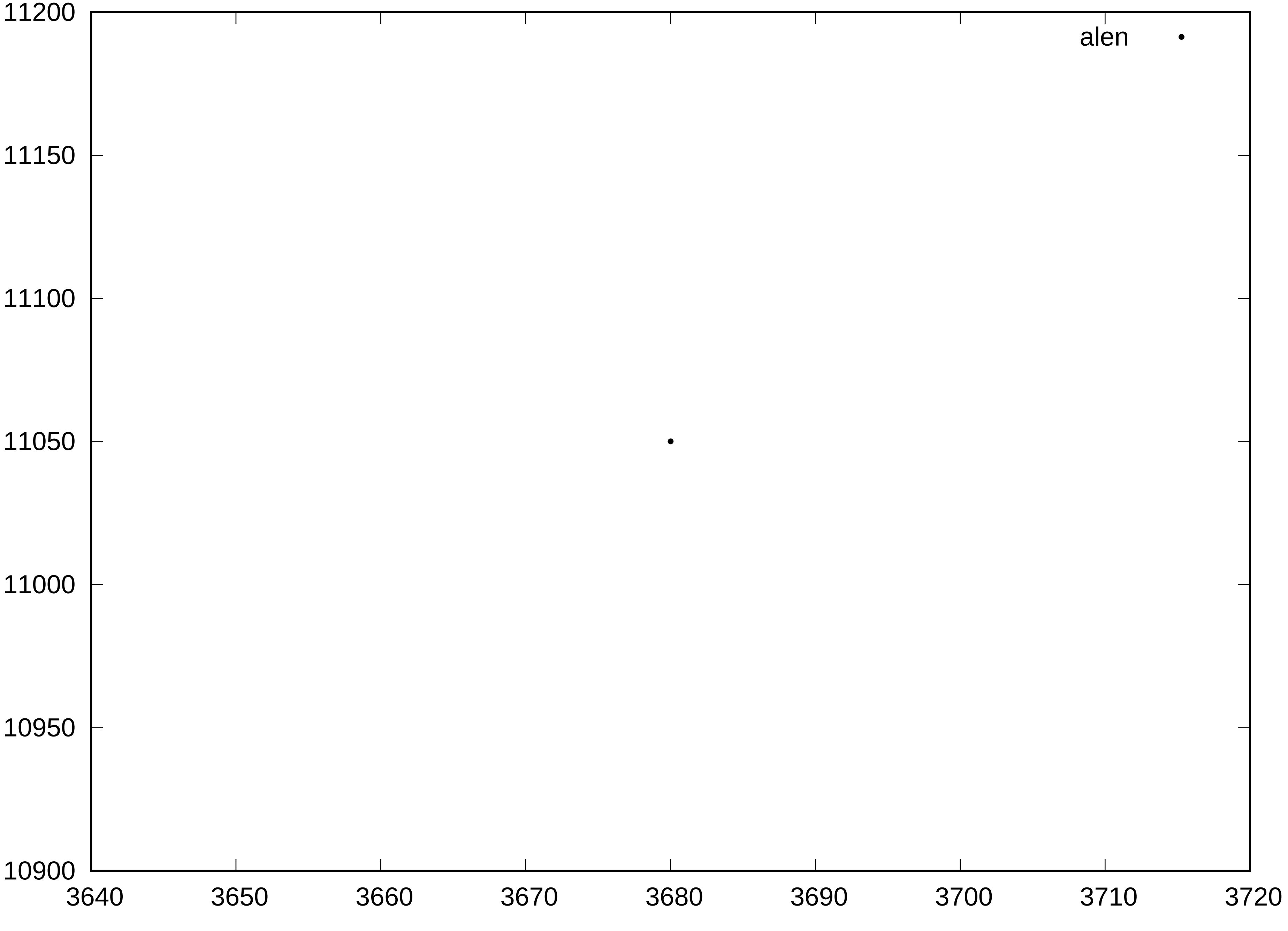
11200
11150
11100
11050
11000
10950
10900
3640
3650
3660
3670
3680
3690
3700
3710
3720
alen

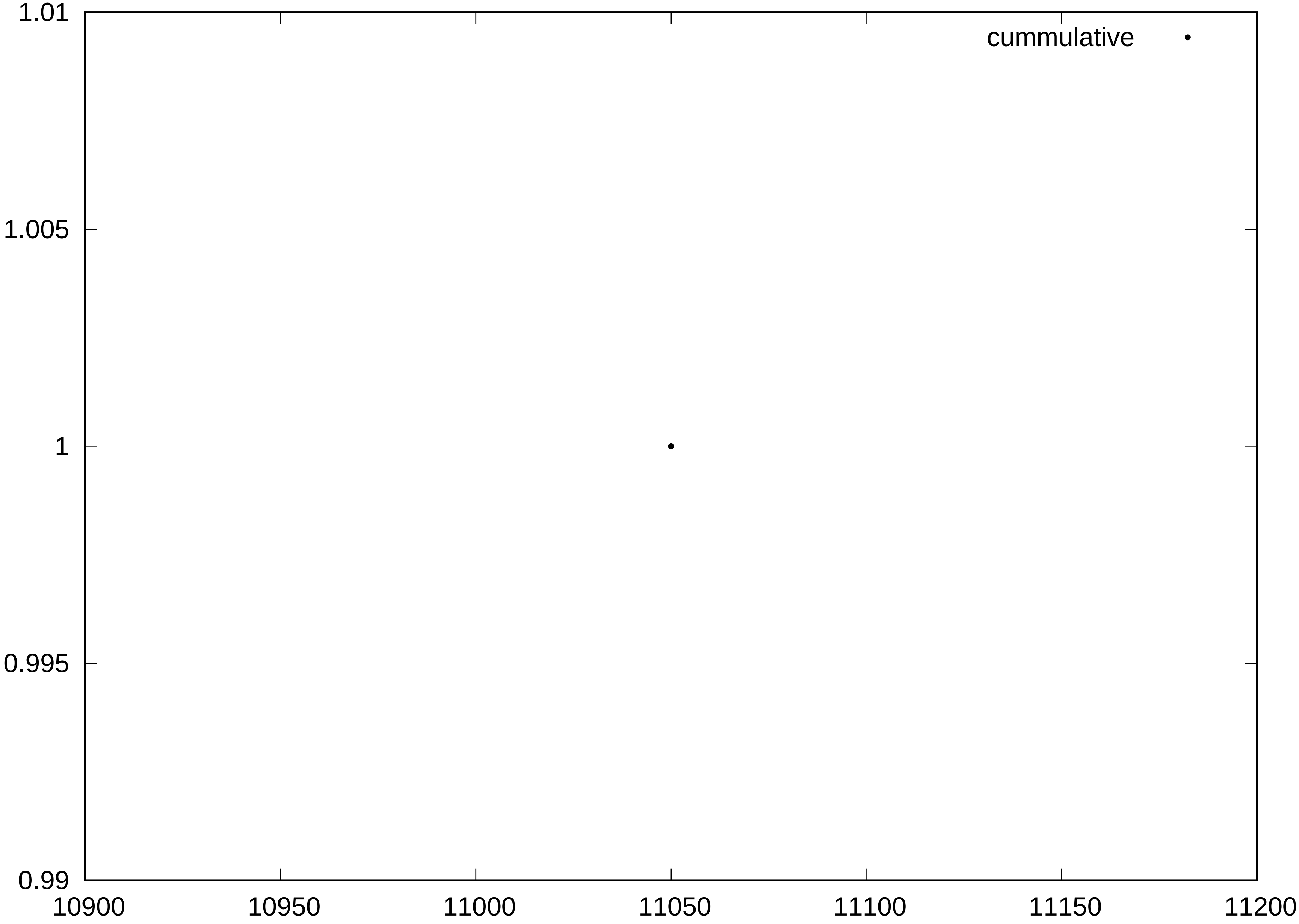

cummulative
1.01
1.005
1
0.995
0.99
10900
10950
11000
11050
11100
11150
11200

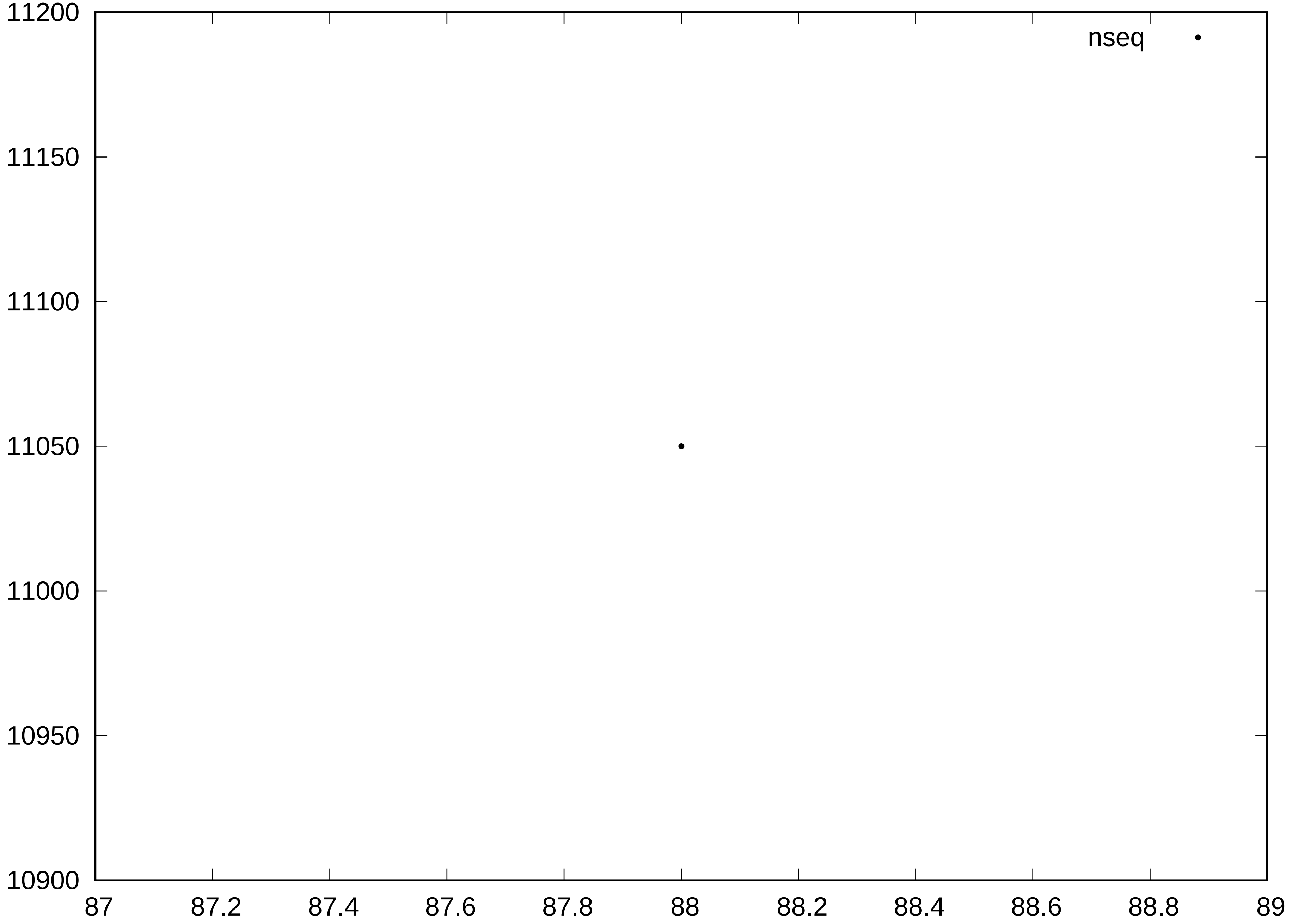

11200
11150
11100
11050
11000
10950
10900
87
87.2
87.4
87.6
87.8
88
88.2
88.4
88.6
88.8
89
nseq

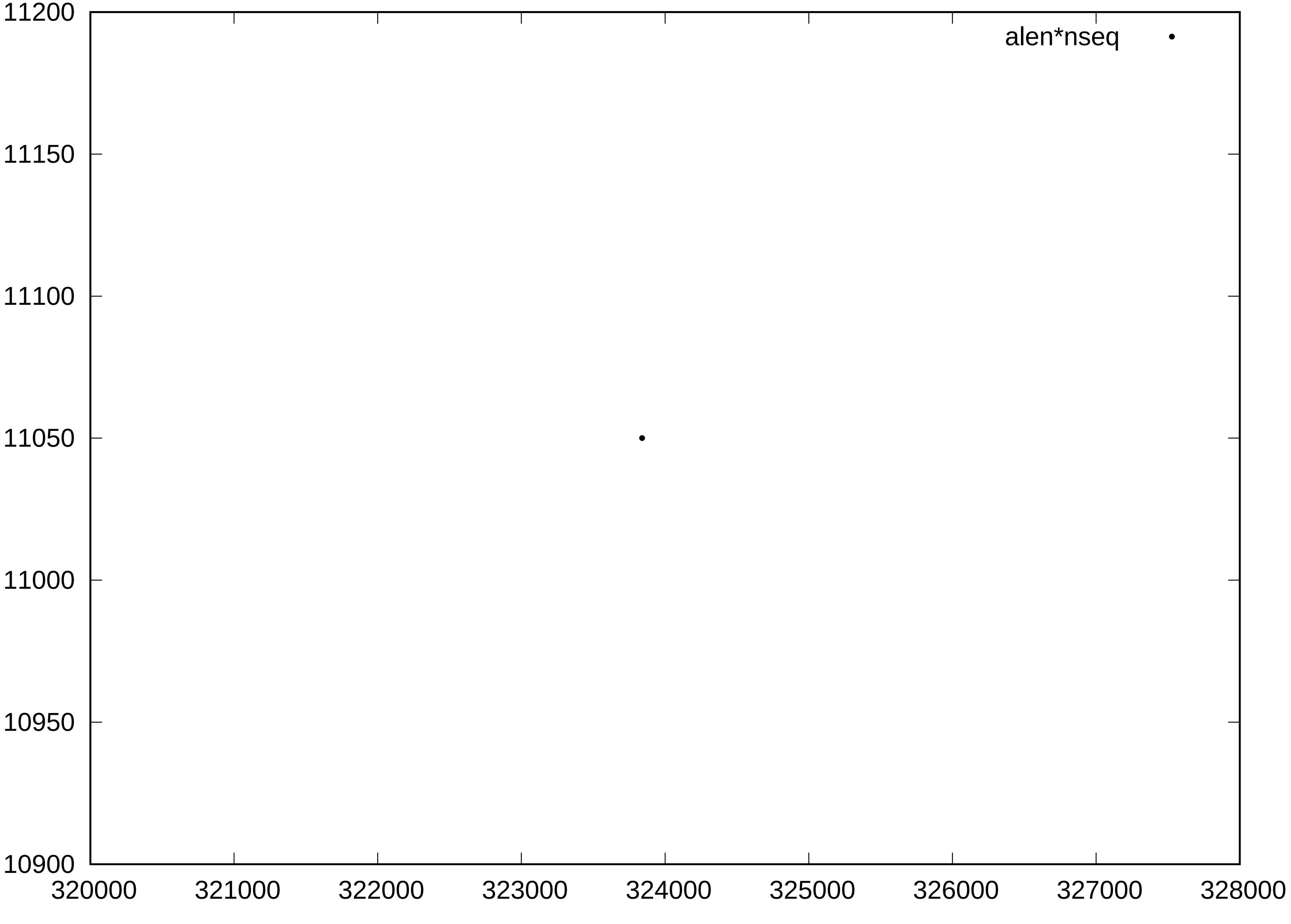
alen*nseq
11200
11150
11100
11050
11000
10950
10900
320000
321000
322000
323000
324000
325000
326000
327000
328000
